# Accuracies of genomically estimated breeding values from pure-breed and across-breed predictions in Australian beef cattle

**DOI:** 10.1186/s12711-014-0061-9

**Published:** 2014-10-24

**Authors:** Vinzent Boerner, David J Johnston, Bruce Tier

**Affiliations:** Animal Genetics and Breeding Unit, University of New England, Armidale, 2351 NSW Australia

## Abstract

**Background:**

The major obstacles for the implementation of genomic selection in Australian beef cattle are the variety of breeds and in general, small numbers of genotyped and phenotyped individuals per breed. The Australian Beef Cooperative Research Center (Beef CRC) investigated these issues by deriving genomic prediction equations (PE) from a training set of animals that covers a range of breeds and crosses including Angus, Murray Grey, Shorthorn, Hereford, Brahman, Belmont Red, Santa Gertrudis and Tropical Composite. This paper presents accuracies of genomically estimated breeding values (GEBV) that were calculated from these PE in the commercial pure-breed beef cattle seed stock sector.

**Methods:**

PE derived by the Beef CRC from multi-breed and pure-breed training populations were applied to genotyped Angus, Limousin and Brahman sires and young animals, but with no pure-breed Limousin in the training population. The accuracy of the resulting GEBV was assessed by their genetic correlation to their phenotypic target trait in a bi-variate REML approach that models GEBV as trait observations.

**Results:**

Accuracies of most GEBV for Angus and Brahman were between 0.1 and 0.4, with accuracies for abattoir carcass traits generally greater than for live animal body composition traits and reproduction traits. Estimated accuracies greater than 0.5 were only observed for Brahman abattoir carcass traits and for Angus carcass rib fat. Averaged across traits within breeds, accuracies of GEBV were highest when PE from the pooled across-breed training population were used. However, for the Angus and Brahman breeds the difference in accuracy from using pure-breed PE was small. For the Limousin breed no reasonable results could be achieved for any trait.

**Conclusion:**

Although accuracies were generally low compared to published accuracies estimated within breeds, they are in line with those derived in other multi-breed populations. Thus PE developed by the Beef CRC can contribute to the implementation of genomic selection in Australian beef cattle breeding.

## Background

Genomic selection (GS) has been introduced into breeding schemes of many livestock species [1-3]. The advantages of GS compared to conventional breeding schemes can be summarised as: (i) shortening the generation interval because genomically estimated breeding values (GEBV) can be calculated early in life, (ii) estimation of GEBV for all genotyped individuals of a species/breed for difficult to measure traits given a prediction equation (PE) that has been derived from a related population and (iii) increased accuracy of estimated breeding values for lowly heritable traits [2,4]. Since in beef cattle breeding, selection candidates usually have some of their own performance records before the selection decision is made, the generation interval is usually not a constraint for the genetic progress. Thus, advantages (ii) and (iii) are the key improvements for Australian beef cattle breeding schemes expected from the implementation of GS [5]. In the dairy industry, several conditions have facilitated the implementation of GS: (1) a large number of phenotypes is collected routinely; (2) wide-spread use of artificial insemination facilitates the use of highly accurate conventionally estimated sire breeding values as pseudo-phenotypes; and (3) large breeding organisations can bear the initial cost of genotyping. Currently, these conditions are not met in the Australian beef cattle industry. On the contrary, the Australian beef industry is made up of a large number of breeds and crosses (both *Bos taurus* and *Bos indicus*), breeding organisations are rather small, and records of economically important traits on live animals or carcasses are usually expensive to measure and limited in number, as are the genotypes of phenotyped individuals [5]. This situation is reflected in low numbers of genotyped individuals which are generally not sufficient to calculate accurate within-breed GEBV.

A possible approach to make GS feasible for breeds with a small number of genotypes and phenotypes is the derivation of PE that allow prediction of GEBV across breeds [4,6]. This derivation is usually done on a mixed breed training population that contains individuals of all targeted breeds. Thus, the number of genotyped individuals in the reference population might exceed the total number of genotyped individuals in any single breed, which may allow for a higher power to detect single nucleotide polymorphisms (SNPs) in strong linkage disequilibrium (LD) with trait coding quantitative trait loci (QTL). However, whether all or only the small breeds gain from this approach depends on the proportions of each breed in the training population [4]. The across-breed-prediction approach was followed by the Australian Beef Cooperative Research Center (www.beefcrc.com, Beef CRC), which derived PE on a pooled training population of genotyped individuals from eight different cattle breeds and on different cross-breed and pure-breed subsets of this pooled set, in which the genotyped individuals originated from Australian populations of Angus, Murray Grey, Shorthorn, Hereford, Brahman, Belmont Red, Santa Gertrudis, Tropical Composite breeds, and F1 crosses of Brahman with Limousin, Charolais, Angus, Shorthorn and Hereford breeds [7].

Beef CRC PE were derived for the commercial animal breeding sector. The value of the PE for breeders depends on the accuracy of the resulting GEBV. This accuracy is the proportion of the additive genetic variance of the focused phenotypic trait, estimated in the commercial seed stock population, that is explained by the GEBV. Common approaches to assess the accuracy and the PE consist in subdividing of the training data and a subsequent n-fold cross-validation, or the derivation of the PE in generation *i* and the derivation of the accuracy in generation *i*+*n*,*n*≥1 [7-11]. However, in both cases, the accuracy is usually calculated as a product moment correlation, sometimes scaled by some value. Whether accuracies obtained in this way are also achievable in the commercial seed stock population depends on a variety of factors such as the genetic distance between the commercial and the training population [12] and the sample size of the training population. Another approach to obtain an estimator of the proportion of the additive genetic variance in the seed stock population explained by the GEBV is to apply PE to genotyped seed stock individuals, model the resulting GEBV as trait observations in a bi-variate approach together with their phenotypic target trait and assess the co-variances by restricted maximum likelihood (REML) or Gibbs sampling [13-16]. This approach accounts for various sources of bias in parameter estimation, including genetic trends, relatedness between individuals, inbreeding and differences in accuracy of EBV. In addition, the genetic correlation between the phenotypic target trait and the GEBV that is obtained this way is an indispensable part of the blending of EBV with GEBV.

The aim of this work was to determine whether Beef CRC PE derived within and across breeds facilitate the introduction of GS in the Australian commercial beef cattle seed stock herds. For this purpose, GEBV were calculated for genotyped seed stock animals of Australian Angus, Limousin and Brahman breeds, thus, subsets of those populations Beef CRC PE have been derived for, and their accuracy was assessed as the genetic correlation to their phenotypic target trait in a bi-variate REML approach.

## Methods

### Genomically estimated breeding values

#### Prediction equations

The assembly of the training population, genotyping of training individuals and PE derivation were not part of this project. For a detailed description of the PE derivation and the size, breed composition and animal characteristics of the training population see [7].

However, in short, PE supplied to the authors were derived within the Beef CRC on 800K Illumina HD Bovine genotypes in a 5-fold cross-validation genomic best linear unbiased prediction (GBLUP) approach with phenotypic records as response variables [7]. PE were developed for the following traits: post-weaning live weight (g.WW), live weight on feedlot entry (g.YW), live weight on feedlot exit (g.FW), carcass rib fat (g.CRIB), carcass P8 fat (g.CP8), carcass intra-muscular fat (g.CIMF) and carcass weight (g.CWT). For a list of GEBV and their abbreviations see Table 1. Genotyped training animals originated from Australian populations of the Angus, Murray Grey, Shorthorn, Hereford, Brahman, Belmont Red, Santa Gertrudis, Tropical Composite breeds, and F1 crosses of the Brahman breed with the Limousin, Charolais, Angus, Shorthorn and Hereford breeds, and included cows, steers and bulls. For each of the above traits, PE were derived on four sets of individuals: all genotyped animals across breeds (ALL), Angus only (ANGUS), *Bos taurus* only (Angus, Murray Grey, Hereford, Shorthorn) (BOSTAURUS) and Brahman only (BRAHMAN).

**Table 1:** **GEBV and phenotypic traits and the used trait abbreviations**

**Trait**	**Abbreviation**
GEBV post-weaning live weight	g.WW
GEBV live weight at feedlot period start	g.YW
GEBV live weight at feedlot period end	g.FW
GEBV scan eye muscle area	g.SEMA
GEBV scan rib fat	g.SRIB
GEBV scan P8 fat	g.SP8
GEBV carcass rib fat	g.CRIB
GEBV carcass p8 fat	g.CP8
GEBV carcass intramuscular fat	g.CIMF
GEBV carcass weight	g.CWT
Phenotypic 200 day weight	p.WW
Phenotypic 400 day weight	p.YW
Phenotypic 600 day weight	p.FW
Phenotypic bull eye muscle area	p.BEMA
Phenotypic heifer/steer eye muscle area	p.HEMA
Phenotypic bull rib fat	p.BRIB
Phenotypic heifer/steer rib fat	p.HRIB
Phenotypic bull P8 fat	p.BP8
Phenotypic heifer/steer P8 fat	p.HP8
Phenotypic carcass rib fat	p.CRIB
Phenotypic carcass P8 fat	p.CP8
Phenotypic carcass intra-muscular fat percentage	p.CIMF
Phenotypic carcass weight	p.CWT

#### Genotypes and genomically estimated breeding values of commercial seed stock animals

The ALL, ANGUS, BOSTAURUS and BRAHMAN PE were applied to genotypes of commercial seed stock animals that originated from Australian populations of the Angus, Limousin and Brahman breeds. None of these genotyped individuals were in the training population. This set of animals will be referred to as “validation set”. For all three breeds, the validation set consisted of widely used sires and animals from the current generation. The numbers of individuals (sires/animals in the current generation) in each breed sample were 1582 (383/1199) for Angus, 782 (368/414) for Limousin, and 400 (108/302) for Brahman. After removing individuals that did not match the breed specific pedigrees, the validation sets consisted of 1487 Angus, 721 Limousin and 400 Brahman individuals. Genotypes of all validation animals were obtained using the Illumina 50K Bead Chip. To apply Beef CRC PE, all genotypes were imputed from 50K to 800K. Imputation was done with a population-based approach [17] using 800K Beef CRC genotypes [7], and 2500 800K genotypes of Limousin, Charolais and Simmental individuals, supplied by the Irish Cattle Breeding Federation (ICBF), as reference genotypes. The population approach was necessary because many of animals in the current generation were not registered at the time of imputation, which made it impossible to exploit possible duo or trio structures in the data because of unknown parents. Finally, GEBV were calculated by applying the above described PE to the animals’ genotypes.

### Compilation of phenotypic datasets

The phenotypic datasets and pedigree data of all three breeds were obtained from databases of their respective breed societies: Angus Australia, Australian Limousin Breeders’ Society and Australian Brahman Breeders’ Association. All phenotypic data were adjusted for systematic effects as described in [18]. For all traits and breeds, the number of records in the phenotypic datasets exceeded those of the GEBV datasets. For Angus, some individuals that were used in the training population were also part of the phenotypic datasets (see Table 2).

**Table 2:** **Parameters of phenotypic traits**

**Trait** ^**1**^	**Angus**				**Limousin**			**Brahman**		
	**N** ^**2**^	**n** ^**3**^	$\overline {\mathbf {x}}^{4}$	***s*** ^**5**^	**N**	$\overline {x}$	**s**	**N**	$\overline {x}$	**s**
p.WW	120928	73	252.3	38.6	83210	239.4	43.6	145558	210.4	39.7
p.YW	81428	39	383.6	71.5	60044	380.0	79.9	67115	280.9	84.7
p.FW	114170	54	535.2	103.5	38545	521.8	115.7	70955	393.6	111.8
p.BEMA	88265	0	83.0	10.8	3450	101.8	14.4	6655	74.6	19.5
p.HEMA	101221	250	62.9	9.9	3237	75.2	14.1	4494	53.6	11.7
p.BRIB	88256	0	3.7	1.5	3381	2.9	1.3	6097	3.8	2.5
p.HRIB	101360	289	5.3	2.3	3229	3.4	1.8	4018	3.4	2.0
p.BP8	88087	0	4.7	2.0	3443	3.4	1.7	6292	5.3	3.4
p.HP8	101530	289	6.8	3.2	3254	4.4	2.6	4184	5.3	3.2
p.CRIB	1203	573	12.3	5.5	-	-	-	1486	7.7	3.7
p.CP8	2183	0	21.0	6.4	1057	11.2	4.5	1575	14.0	5.1
p.CIMF	2822	0	7.2	2.6	986	3.4	1.5	1584	2.8	1.3
p.CWT	3839	0	400.4	47.4	1082	303.2	67.7	-	-	-

^1^for trait abbreviations see Table 1, ^2^number of phenotypic observations, ^3^number of Angus individuals with a phenotypic record in this data set which have been used for deriving ANGUS, BOSTAURUS and ALL prediction equation, ^4^mean, ^5^standard deviation. Note that no Limousin individuals were used in the training set and all phenotyped Brahman individuals which where part of the training set were excluded from the analysis.

Phenotypic traits included in the analysis were 200-day weight (p.WW), 400-day weight (p.YW), 600-day weight (p.FW), bull’s scan eye muscle area (p.BEMA), heifer’s scan eye muscle area (p.HEMA), bull’s scan rib fat (p.BRIB), heifer’s scan rib fat (p.HRIB), bull’s scan P8 fat (p.BP8), heifer’s scan P8 fat (p.HP8), carcass rib fat (p.CRIB), carcass P8 fat (p.CP8), carcass intra-muscular fat (p.CIMF) and carcass weight (p.CWT). Note that not all traits were available for each breed. For a list of phenotypic traits and their abbreviations see Table 1.

Contemporary groups were formed as defined in [18] but all single record groups were deleted. For p.WW, p.YW and p.FW, records were excluded if the sire, dam, maternal grandsire or embryo transfer recipient dam was unknown. Multiple records of these traits were also deleted except for the first. In order to decrease the computational demand, the number of Angus records for p.WW and p.YW was further reduced as follows: records were kept only if the recorded individual was part of the validation set, or was a direct progeny of an individual in the validation set, or was in a contemporary group with an individual that belonged to one of the latter two groups.

### Estimation of the variance components

Variances and variance ratios were obtained from bi-variate REML analysis for which each phenotypic trait was analysed in conjunction with its assigned GEBV. The fitted model for p.WW, p.YW and p.FW and their respective GEBV was:

(1) $$ \begin{array}{ll} \left(\begin{array}{l} \mathbf{y} \\ \mathbf{y}_{\text{\textbf{GEBV}}}\\ \end{array}\! \right) &= \left(\begin{array}{cc} \textbf{X} & \text{0} \\ \text{0} & \textbf{1} \\ \end{array} \right) \left(\begin{array}{l} \mathbf{b}_{\mathbf{p}} \\ \mathbf{b}_{\mathbf{g}} \\ \end{array}\! \right) + \left(\begin{array}{cccc} \mathbf{Z}_{\mathbf{d}} & \text{0} & \mathbf{Z}_{\mathbf{m}} & \mathbf{Z}_{\mathbf{p}} \\ \text{0} & \mathbf{Z}_{\mathbf{g}} & \text{0} & \text{0}\\ \end{array}\! \right) \left(\begin{array}{l} \mathbf{u}_{\mathbf{d}} \\ \mathbf{u}_{\mathbf{g}} \\ \mathbf{u}_{\mathbf{m}} \\ \mathbf{p} \\ \end{array}\! \right)\\ &\quad + \left(\begin{array}{l} \mathbf{e}_{\mathbf{p}} \\ \mathbf{e}_{\mathbf{g}} \\ \end{array} \right) \end{array}  $$

where **y**, **y**_**GEBV**_, **b**_**p**_, **b**_**g**_, **u**_**g**_, **u**_**d**_, **u**_**m**_, **p**, **e**_**p**_ and **e**_**g**_ are vectors of phenotypic observations, GEBV, fixed effects of the phenotypic trait, the mean of the GEBV, random direct additive genetic effects of the GEBV, random direct additive genetic, random maternal additive genetic, random maternal environmental and random residual effects of the phenotypic trait and random residual effects of the GEBV, respectively. **X**, **Z**_**d**_, **Z**_**g**_, **Z**_**m**_ and **Z**_**p**_ are incidence matrices relating the effects to their phenotypic observations or GEBV, respectively, and **1** is a vector of 1s. Random effects in the model were assumed to be multivariate normally distributed with

(2) $$ {\small\begin{aligned} \left(\! \begin{array}{c} \mathbf{u}_{\mathbf{d}} \\ \mathbf{u}_{\mathbf{g}} \\ \mathbf{u}_{\mathbf{m}} \\ \mathbf{p} \\ \mathbf{e}_{\mathbf{p}} \\ \mathbf{e}_{\mathbf{g}} \\ \end{array}\! \right)\! \sim \text{N}\! \left(\left[\! \begin{array}{c} 0 \\ 0 \\ 0 \\ 0 \\ 0 \\ 0 \\ \end{array}\! \right]\!, \left[\!\! \begin{array}{ccccccc} \mathbf{A}\sigma_{\text{a}}^{2} & \mathbf{A}\sigma_{\text{a,g}} & \mathbf{A}\sigma_{\text{a,m}} & \text{0} & \text{0} & \text{0} \\ \mathbf{A}\sigma_{\text{a,g}} & \mathbf{A}\sigma_{\text{g}}^{2} & \mathbf{A}\sigma_{\text{g,m}}& \text{0} & \text{0} & \text{0}\\ \mathbf{A}\sigma_{\text{a,m}} & \mathbf{A}\sigma_{\text{g,m}}& \mathbf{A}\sigma_{\text{m}}^{2} & \text{0} & \text{0} & \text{0}\\ 0 & 0 & 0 & \mathbf{I}{\sigma_{p}^{2}} & 0 & 0\\ 0 & 0 & 0 & 0 & \mathbf{I}\sigma_{\text{e}_{\text{p}}}^{2} & \mathbf{I}\sigma_{\text{e}_{\text{p,g}}}\\ 0 & 0 & 0 & 0 & \mathbf{I}\sigma_{\text{e}_{\text{p,g}}} &\mathbf{I}\sigma_{\text{e}_{\text{g}}}^{2}\\ \end{array}\! \right] \right)\!, \end{aligned}}  $$

where **A** is the numerator relationship matrix constructed such that every individual with a phenotypic or GEBV observation had at least three generations of ancestors in the pedigree if available, and **I** is an identity matrix. $\sigma _{\text {a}}^{2}$ is the variance of the direct additive genetic effect of the phenotypic trait, $\sigma _{\text {g}}^{2}$ is the variance of the direct additive genetic effect of the GEBV, $\sigma _{\text {m}}^{2}$ is the variance of the maternal additive genetic effect of the phenotypic trait, *σ*_a,m_ is the covariance between the direct additive genetic effect of the phenotypic trait and the maternal additive genetic effect of the phenotypic trait, *σ*_a,g_ is the covariance between the direct additive genetic effect of the phenotypic trait and the direct additive genetic effect of the GEBV, *σ*_g,m_ is the covariance between the direct additive genetic effect of the GEBV and the maternal additive genetic effect of the phenotypic trait, $\sigma _{\text {p}}^{2}$ is the variance of the maternal permanent environmental effect, $\sigma _{\text {e}_{\text {p}}}^{2}$ is the variance of the residual effect of the phenotypic trait, $\sigma _{\text {e}_{\text {g}}}^{2}$ is the variance of the residual effect of the GEBV, and $\phantom {\dot {i}\!}\sigma _{\text {e}_{\text {p,g}}}$ is the co-variance of the residual effect of the GEBV and the residual effect of the phenotypic trait.

The fitted bi-variate model for all other phenotypic traits and their respective GEBV was:

(3) $$ \begin{array}{ll} \left(\begin{array}{l} \mathbf{y} \\ \mathbf{y}_{\text{\textbf{GEBV}}} \\ \end{array}\! \right) &= \left(\begin{array}{cc} \mathbf{X} & 0 \\ 0 & 1 \\ \end{array} \right) \left(\begin{array}{l} \mathbf{b}_{\mathbf{p}} \\ \mathbf{b}_{\mathbf{g}} \\ \end{array} \right) + \left(\begin{array}{cc} \mathbf{Z}_{\mathbf{d}} & 0 \\ 0 & \mathbf{Z}_{\mathbf{g}} \\ \end{array} \right) \left(\begin{array}{l} \mathbf{u}_{\mathbf{d}} \\ \mathbf{u}_{\mathbf{g}} \\ \end{array} \right)\\ &\quad + \left(\begin{array}{l} \mathbf{e}_{\mathbf{p}} \\ \mathbf{e}_{\mathbf{g}} \\ \end{array} \right) \end{array},  $$

with random effects assumed to be multivariate normally distributed with

(4) $$ \left(\begin{array}{c} \mathbf{u}_{\mathbf{d}} \\ \mathbf{u}_{\mathbf{g}} \\ \mathbf{e}_{\mathbf{p}} \\ \mathbf{e}_{\mathbf{g}} \\ \end{array} \right) \sim \text{N} \left(\left[ \begin{array}{c} 0 \\ 0 \\ 0 \\ 0 \\ \end{array} \right], \left[ \begin{array}{ccccccc} \mathbf{A}\sigma_{\text{a}}^{2} & \mathbf{A}\sigma_{\text{a,g}} & 0 & 0 \\ \mathbf{A}\sigma_{\text{a,g}} & \mathbf{A}\sigma_{\text{g}}^{2} & 0 & 0\\ 0 & 0 &\mathbf{I}\sigma_{\text{e}_{\text{p}}}^{2} & \mathbf{I}\sigma_{\text{e}_{\text{p,g}}}\\ 0 & 0 & \mathbf{I}\sigma_{\text{e}_{\text{p,g}}} & \mathbf{I}\sigma_{\text{e}_{\text{p}}}^{2} \\ \end{array} \right] \right).  $$

For phenotypic traits, contemporary group was the only fixed effect, and for GEBV only the mean was fitted as a fixed effect. Note that the residual covariance was fitted only for the combinations of GEBV and phenotypic traits where a subset of the individuals had observations on both, the GEBV and the phenotypic trait.

### Software

Imputation was done with Beagle [17] without exploiting any parent-offspring pair/parent-offspring trio structure. The number of iterations in Beagle was set to 30. Pre- and post-analysis data manipulation was done with R [19] and Sweave [20]. REML analyses were carried out with WOMBAT [21].

## Results

### Raw data

Table 2 summarises the results for all phenotypic traits across breeds for the following parameters: number of observations, mean, standard deviation and number of animals that are in common between the phenotypic dataset and the training population. The number of observations for growth traits exceeded those for the difficult and expensive-to-measure carcass traits for all three breeds. Large numbers of records for live animal ultrasound scan traits were available for the Angus breed only, whereas for the Brahman and Limousin breeds records of these traits were almost as limited as those for carcass traits. An overlap between the phenotypic dataset and the training dataset was found for the Angus breed only, which means that in this case the datasets were not totally independent. However, only for p.CRIB, could the proportion of training individuals used in the phenotypic dataset (573 of 1203 phenotypic observations) have caused an upward biased accuracy. For all other traits, this proportion was zero or negligible due to more phenotypic records and less training individuals in the phenotypic dataset. As mentioned above, none of the genotyped validation animals were used in the training population. However, the mean, minimum and maximum relationships between the validation individuals and those training individuals of the same breed, based on a pedigree constructed for these animals three generations back, were equal to 0.014, 0.0 and 0.57 for Angus, and 0.008, 0.0 and 0.57 for Brahman. Note that the mean relationship includes only training individuals of the same breed as the target population. Thus, for mixed-breed training populations, this number is even smaller when all training individuals are included, and if the training population and the target population represent different breeds, all three parameters are equal to 0.

### Heritabilities of genomically estimated breeding values

Table 3 summarises heritabilities (h^2^) and their standard errors for all GEBV. Across traits and PE high h^2^ of almost 1 with the lowest standard errors were found consistently for the Angus breed only. For the Brahman breed, in most cases the h^2^ values were below 0.9. Across traits, the lowest h^2^ values were always estimated for the GEBV calculated from the ANGUS PE, followed by those from the BOSTAURUS PE and BRAHMAN PE. The highest h^2^ were almost exclusively estimated for ALL PE GEBV, except for g.WW which was below 0.9. In most cases, the standard errors of h^2^ for the Brahman breed GEBV were above 0.1, and therefore about five times as large as those for Angus, which reflects the size of the Brahman sample. The lowest h^2^ across traits and PE were found for the Limousin breed with most values below 0.6 and the lowest estimates equal to 0.42 for g.SRIB from ALL PE. In contrast to Brahman, no generally superior or inferior PE could be identified for Limousin. Heritabilities from the uni-variate analysis were not different to those from the bi-variate analysis (results not shown).

**Table 3:** **Heritabilities (upper) and standard errors (lower) of GEBV**

**Trait** ^**1**^	**Angus (n** ^**2**^ ** = 1487)**				**Limousin (n = 721)**				**Brahman (n = 400)**			
	**ALL** ^**3**^	**An** ^**4**^	**Bt** ^**5**^	**Br** ^**6**^	**ALL**	**An**	**Bt**	**Br**	**ALL**	**An**	**Bt**	**Br**
g.WW	0.95	1.00	0.98	0.99	0.61	0.50	0.59	0.65	0.71	0.64	0.71	0.84
g.YW	0.96	0.99	0.96	0.99	0.56	0.46	0.42	0.70	0.99	0.68	0.74	0.96
g.FW	0.96	0.99	0.98	1.00	0.43	0.58	0.57	0.58	0.97	0.77	0.86	0.87
g.SEMA	0.97	0.98	0.99	0.97	0.76	0.78	0.47	0.59	0.92	0.88	0.86	0.89
g.SRIB	1.00	1.00	1.00	1.00	0.42	0.49	0.46	0.58	1.00	0.85	0.99	0.99
g.SP8	0.97	0.97	0.97	0.96	0.56	0.57	0.62	0.58	0.97	0.78	0.88	0.87
g.CRIB	0.98	1.00	0.99	1.00	-	-	-	-	0.96	0.96	1.00	0.99
g.CP8	0.97	0.99	0.96	1.00	0.46	0.66	0.54	0.66	0.96	0.94	0.81	0.87
g.CIMF	0.99	1.00	1.00	1.00	0.65	0.54	0.76	0.59	0.90	0.83	0.92	0.92
g.CWT	0.95	1.00	0.93	1.00	0.47	0.50	0.56	0.60	-	-	-	-
g.WW	0.03	0.02	0.03	0.03	0.10	0.10	0.10	0.09	0.15	0.14	0.12	0.13
g.YW	0.03	0.03	0.03	0.03	0.10	0.10	0.10	0.09	0.11	0.14	0.13	0.11
g.FW	0.03	0.02	0.03	0.03	0.11	0.10	0.10	0.09	0.11	0.13	0.12	0.12
g.SEMA	0.03	0.03	0.03	0.03	0.07	0.07	0.09	0.09	0.11	0.13	0.12	0.12
g.SRIB	0.03	0.03	0.03	0.03	0.10	0.10	0.11	0.09	0.08	0.13	0.11	0.09
g.SP8	0.03	0.03	0.03	0.04	0.10	0.10	0.10	0.10	0.10	0.14	0.13	0.12
g.CRIB	0.03	0.03	0.03	0.03	-	-	-	-	0.10	0.10	0.10	0.09
g.CP8	0.03	0.03	0.03	0.03	0.10	0.09	0.10	0.08	0.09	0.11	0.13	0.09
g.CIMF	0.02	0.02	0.02	0.03	0.09	0.09	0.08	0.09	0.10	0.13	0.11	0.11
g.CWT	0.04	0.02	0.04	0.03	0.10	0.10	0.10	0.09	-	-	-	-

^1^for trait abbreviations see Table 1, ^2^number of individuals with a GEBV, ^3^prediction equation derived on a mixed-breed training population, ^4^prediction equation derived on a training population of Angus individuals, ^5^prediction equation derived on a training population of *Bos taurus* individuals, ^6^prediction equation derived on a training population of Brahman individuals, - : for Limousin cattle no data were available.

### Genetic correlations between GEBV and phenotypic traits

Table 4 summarises the genetic correlations (r_g_) between GEBV and phenotypic traits for the Australian Angus breed. The highest r_g_ (0.53) was found for p.CRIB :g.CRIB derived from BOSTAURUS PE, the lowest (-0.01) for p.BP8 :g.SP8 derived from BRAHMAN PE, but most values were below 0.2. Across all traits, ALL PE and BOSTAURUS PE yielded the highest r_g_ followed by ANGUS and BRAHMAN PE, where the ALL PE results almost mirrored those from ANGUS and BOSTAURUS PE. BRAHMAN PE was inferior for carcass traits, whereas for growth traits (except p.FW :g.FW) differences between r_g_ of GEBV from different PE were small. As a result of the number of phenotypic observations, standard errors of r_g_ for growth traits were below 0.1, but much larger for carcass traits for which fewer data were available.

**Table 4:** **Correlation|** ***standard error*** **between the direct additive genetic component of the phenotypic trait and GEBV from different prediction equations for Australian Angus Cattle**

**Phenotypic** ^**1**^	**GEBV** ^**1**^	**ALL** ^**2**^	**ANG** ^**3**^	**BT** ^**4**^	**BRA** ^**5**^
p.WW	g.WW	0.09|*0.06*	0.07|*0.05*	0.10|*0.06*	0.11|*0.06*
p.YW	g.YW	0.08|*0.06*	0.09|*0.06*	0.09|*0.06*	0.14|*0.06*
p.FW	g.FW	0.19|*0.05*	0.18|*0.05*	0.21|*0.05*	0.11|*0.06*
p.BEMA	g.SEMA	0.16|*0.06*	0.18|*0.06*	0.16|*0.06*	0.01|*0.06*
p.HEMA	g.SEMA	0.15|*0.05*	0.10|*0.05*	0.13|*0.05*	0.09|*0.06*
p.BRIB	g.SRIB	0.26|*0.06*	0.25|*0.06*	0.25|*0.05*	0.09|*0.06*
p.HRIB	g.SRIB	0.20|*0.05*	0.19|*0.05*	0.20|*0.05*	0.05|*0.05*
p.BP8	g.SP8	0.25|*0.06*	0.27|*0.06*	0.25|*0.06*	-0.01|*0.06*
p.HP8	g.SP8	0.21|*0.05*	0.23|*0.05*	0.21|*0.05*	0.02|*0.05*
p.CRIB	g.CRIB	0.51|*0.21*	0.36|*0.17*	0.53|*0.21*	0.12|*0.35*
p.CP8	g.CP8	0.36|*0.20*	0.27|*0.17*	0.34|*0.19*	0.12|*0.23*
p.CIMF	g.CIMF	0.33|*0.12*	0.29|*0.12*	0.36|*0.13*	0.00|*0.17*
p.CWT	g.CWT	0.25|*0.15*	0.30|*0.12*	0.25|*0.14*	-0.00|*0.17*

^1^for trait abbreviations see Table 1, ^2^prediction equation derived on a mixed-breed training population, ^3^prediction equation derived on a training population of Angus individuals, ^4^prediction equation derived on a training population of *Bos taurus* individuals, ^5^prediction equation derived on a training population of Brahman individuals.

For the Limousin breed, r_g_ varied more than for the Angus breed, and their standard errors were much greater (see Table 5). Across traits and PE r_g_ varied from 0.63 to -0.69 for p.CP8 :g.CP8 estimated from BOSTAURUS PE and BRAHMAN PE, respectively. No clear pattern regarding superior or inferior PE could be identified because r_g_ varied considerably within traits across PE. For example, r_g_ of p.WW :g.WW was -0.02 from ALL PE, 0.22 from ANGUS PE, 0.08 from BOSTAURUS PE and -0.03 from BRAHMAN PE.

**Table 5:** **Correlation|** ***standard error*** **between the direct additive genetic component of the phenotypic trait and GEBV from different prediction equations for Australian Limousin cattle**

**Phenotypic** ^**1**^	**GEBV** ^**1**^	**ALL** ^**2**^	**ANG** ^**3**^	**BT** ^**4**^	**BRA** ^**5**^
p.WW	g.WW	-0.01|*0.09*	0.22|*0.10*	0.08|*0.10*	-0.03|*0.09*
p.YW	g.YW	-0.15|*0.10*	0.02|*0.11*	0.06|*0.11*	-0.11|*0.09*
p.FW	g.FW	-0.03|*0.11*	-0.01|*0.10*	-0.03|*0.10*	0.06|*0.10*
p.BEMA	g.SEMA	0.28|*0.14*	-0.29|*0.13*	-0.01|*0.19*	0.01|*0.16*
p.HEMA	g.SEMA	-0.29|*0.16*	-0.59|*0.10*	-0.25|*0.17*	-0.25|*0.16*
p.BRIB	g.SRIB	-0.40|*0.19*	-0.14|*0.17*	-0.36|*0.17*	-0.23|*0.16*
p.HRIB	g.SRIB	0.40|*0.13*	-0.38|*0.11*	-0.06|*0.17*	-0.42|*0.11*
p.BP8	g.SP8	-0.13|*0.16*	-0.27|*0.12*	-0.24|*0.13*	-0.20|*0.15*
p.HP8	g.SP8	0.08|*0.16*	0.01|*0.15*	0.02|*0.15*	-0.05|*0.15*
p.CP8	g.CP8	0.08|*0.36*	-0.14|*0.29*	0.63|*0.28*	-0.69|*0.18*
p.CIMF	g.CIMF	0.47|*0.25*	-0.26|*0.30*	-0.04|*0.24*	0.23|*0.30*
p.CWT	g.CWT	0.24|*0.29*	0.06|*0.31*	0.34|*0.28*	0.17|*0.29*

^1^for trait abbreviations see Table 1, ^2^prediction equation derived on a mixed-breed training population, ^3^prediction equation derived on a training population of Angus individuals, ^4^prediction equation derived in a training population of *Bos taurus* individuals, ^5^prediction equation derived on a training population of Brahman individuals.

Table 6 summarises the GEBV r_g_ for Australian Brahman, which varied across traits and PE from 0.7 (p.CRIB :g.CRIB from ALL PE) to -0.5 (p.CIMF :g.CIMF from ANGUS PE). However, negative r_g_ were exclusively found in ANGUS and BOSTAURUS PE. Moreover, results from ALL PE almost mirrored those from BRAHMAN PE, whereas ANGUS and BOSTAURUS PE yielded much smaller or even negative r_g_. Standard errors decreased with the availability of more phenotypic data (low for carcass traits and high for growth traits), and were similar across PE, except for most carcass traits, for which standard errors from ANGUS and BOSTAURUS PE were double those from ALL and BRAHMAN PE.

**Table 6:** **Correlation|** ***standard error*** **between the direct additive genetic component of the phenotypic trait and GEBV from different prediction equations for Australian Brahman cattle**

**Phenotypic** ^**1**^	**GEBV** ^**1**^	**ALL** ^**2**^	**ANG** ^**3**^	**BT** ^**4**^	**BRA** ^**5**^
p.WW	g.WW	0.27|*0.10*	0.07|*0.11*	0.15|*0.10*	0.23|*0.09*
p.YW	g.YW	0.19|*0.10*	0.14|*0.11*	0.14|*0.11*	0.20|*0.09*
p.FW	g.FW	0.20|*0.09*	-0.17|*0.11*	-0.07|*0.10*	0.20|*0.10*
p.BEMA	g.SEMA	-0.08|*0.22*	0.19|*0.26*	-0.24|*0.26*	-0.17|*0.23*
p.HEMA	g.SEMA	-0.04|*0.24*	-0.23|*0.26*	-0.10|*0.27*	-0.06|*0.26*
p.BRIB	g.SRIB	0.45|*0.17*	-0.08|*0.23*	0.01|*0.22*	0.41|*0.17*
p.HRIB	g.SRIB	0.18|*0.22*	-0.29|*0.26*	-0.14|*0.25*	0.19|*0.23*
p.BP8	g.SP8	0.34|*0.20*	-0.08|*0.21*	-0.16|*0.21*	0.24|*0.20*
p.HP8	g.SP8	0.32|*0.21*	0.20|*0.23*	-0.09|*0.23*	0.30|*0.21*
p.CRIB	g.CRIB	0.70|*0.20*	0.10|*0.46*	0.44|*0.43*	0.65|*0.21*
p.CP8	g.CP8	0.57|*0.19*	0.46|*0.30*	0.44|*0.34*	0.34|*0.24*
p.CIMF	g.CIMF	0.56|*0.27*	-0.50|*0.37*	-0.20|*0.41*	0.36|*0.25*

^1^for trait abbreviations see Table 1, ^2^prediction equation derived on a mixed-breed training population, ^3^prediction equation derived an a training population of Angus individuals, ^4^prediction equation derived on a training population of *Bos taurus* individuals, ^5^prediction equation derived on a training population of Brahman individuals.

## Discussion

### Genetic correlations

Across-breed PE were derived by the Beef CRC to facilitate the implementation of GS in the Australian beef cattle industry, which is made difficult by the large number of breeds, small numbers of individuals with genotypes and/or phenotypes per breed and their unequal distribution across breeds, and the widespread use of cross-breeds. It has been proposed that PE derived from large mixed-breed samples may circumvent these problems. Moreover, the power of detection of SNPs in high LD with a QTL that affects the phenotype of interest is expected to increase when mixed-breed data is used [8,22].

Accuracy of GEBV derived from Beef CRC PE in a 5-fold cross-validation approach were published by [7]. Since Beef CRC prediction equations were developed for application in the Australian commercial beef cattle seed stock herds, the aim of this work was to validate accuracies in these herds via a bi-variate REML approach. In addition, estimated parameters are a precondition for blending estimated breeding values with GEBV. Accuracies of GEBV from ALL PE for Australian Angus were calculated as REML genetic correlations between GEBV and their phenotypic target traits, and were found to be considerably different to those given by [7]. For instance cross-validation accuracies of g.WW, g.YW, g.SRIB and g.SP8 for the Angus breed were reported to be equal to 0.27, 0.42, 0.42 and 0.5 respectively, while the values estimated in our study were 0.09, 0.08, 0.26 and 0.25 respectively. On the contrary, cross-validation accuracies for g.SEMA, g.CIMF and g.CWT reported by [7] were equal to those found here (0.15 vs. 0.15, 0.31 vs. 0.33) or lower (0.16 vs. 0.25). However, the standard errors of our results do not allow us to draw an unambiguous conclusion on whether the latter three estimates are significantly different from 0. For the Brahman breed, accuracies published by [7] for g.WW, g.YW and g.SEMA were also considerably higher than those obtained from ALL PE. In contrast, for g.SP8 and g.CIMF, ALL PE yielded higher accuracies (0.34 vs. 0.19, 0.56 vs. 0.27). However, for these GEBV the standard errors do not support the conclusion that accuracies are significantly different from 0. One possible reason for the differences is the genetic distance between our validation dataset and the training dataset. The Beef CRC collection of genotypes started in the early 2000. Thus, the distance between some validation and training genotypes might represent several generations. Moreover, some genotypes were collected from special selection lines [7].

Compared with the range of results published in other studies, the accuracies of GEBV for Australian Angus presented here are generally at the lower end of the range [13,14,23-25]. For example accuracies of GEBV that are commercially available from Igenity (www.igenity.com) for growth traits, carcass marbling and carcass weight in Australian/American Angus were generally higher than 0.4 [23,25]. In contrast, especially for growth traits, our values were lower than 0.1 except the accuracy of g.FW. The same applies to GEBV that are commercially available from Zoetis (www.Zoetis.com) [23,24]. In all the studies cited above, GEBV were evaluated within-breed only, but Beef CRC PE were derived across indicine and taurine breeds. Studies on beef cattle across-breed predictions are limited [14,15], but accuracies of g.CIMF and g.WW reported here were in the same range than those in [15]. However, accuracies of g.YW was ∼ 0.1, whereas results of both the latter citations were between 0.3 and 0.45. Moreover, [14] found an accuracy of g.WW of 0.36, compared to our result of 0.09 from ALL PE. Differences between accuracies obtained from different PE were minor except between the BRAHMAN PE and the other three PE. The small differences in accuracies obtained from the ANGUS and BOSTAURUS PE may result from the *Bos taurus* training set consisting of almost 50% Angus individuals [7]. However, the addition of indicine breeds to the training set, which represented about 60% of the ALL PE training set, had small positive effects on the accuracies of almost all GEBV. In contrast, the BRAHMAN PE performed worst in the Angus breed for most traits, which combined to the results from ALL PE, reinforces the empirical finding that the target breed must be a member of the training population [14]. However, given the high standard errors, in general differences between accuracies obtained from different PE for a given trait were not statistically significant.

Accuracies of GEBV from ALL PE for the Limousin breed reflect that no pure-breed Limousin individuals were part of the training population. Generally, accuracies reported here do not show any consistent pattern within traits across PE. In contrast, accuracies for the American Limousin population from within-breed predictions were equal to ∼0.4, and for yearling weight, they even reached 0.76 [26]. Moreover, accuracies of GEBV predicted from PE derived from a cross-breed population that consisted of only about 7% Limousin genome were between 0.2 and 0.65 depending on the trait [15].

For the Brahman breed, the only pure-breed *Bos indicus* cattle in the training population, the ALL PE yielded the highest accuracies for most GEBV, followed by the BRAHMAN PE, whereas the ANGUS and BOSTAURUS PE yielded negative results in most cases. The poor performance of the BOSTAURUS and ANGUS PE is in line with the poor performance of the BRAHMAN PE in the Angus breed, which reflects the need of having all predicted breeds in the training population. The better performance of the ALL PE compared to the BRAHMAN PE might result from additional information embedded in the LD between certain SNPs and QTL across *Bos taurus* and *Bos indicus* sub-species, in conjunction with a higher power of detection due to an increased training population size [8,22]. However, the standard errors of the accuracies do not allow for a statistically based preference of a certain PE.

### Heritabilities

Low heritabilities of GEBV indicate that our results for the Limousin breed and partly for the Brahman breed may be affected by genotyping errors, pedigree errors or very low relationships between individuals with GEBV. For the Limousin and Brahman breeds, heritabilities varied considerably within traits across PE (e.g. for g.WW 0.5 to 0.65 for Limousin, 0.64 to 0.84 for Brahman). Since GEBV are linear functions of SNP genotypes, and SNP genotypes were the same for all PE, the heritabilities of GEBV for the same trait from different PE were expected to be equal. This assumption holds only if genotypes are obtained without errors, or if genotyping and imputation errors affect all SNPs equally. If some SNPs are more affected by errors than others and the PE weight SNPs differently, the heritabilities of GEBV for a certain trait from different PE may vary although the same animals and genotypes were used. However, this is only expected to be the case when evaluating PE in target populations because poor genotyping/imputation quality of individuals in the training population is accounted for by the prediction equation via altered GEBV accuracy. Thus, if the genotypes of validation animals were affected by imputation errors, accuracies of GEBV may increase as a result of an increased imputation accuracy.

### Estimation of accuracies

Genomic PE are usually derived to implement GS in certain target populations by supplying the PE or GEBV to the breeding organisations. The parameter of paramount interest when evaluating PE or GEBV is the proportion of the additive genetic variance of the phenotypic target trait in the target population explained by the GEBV, where the square root of this parameter is the accuracy of the GEBV. From the perspective of the breeding organisation, this parameter can be obtained either by using the accuracy generated during the process of generating the PE, which assumes that this accuracy is equal to a variance ratio, or by re-estimating this parameter in the target population. Using the accuracy from the PE generation process bears the risk of assuming the GEBV to be more accurate than they actually are if the genetic link to the training population is insufficient [12], or if the training population sample size does not reflect the genetic variability in the target population because parameters estimated in the training population may not be valid in the target population. This problem can be circumvented by re-estimating the accuracy in the target population. However, accuracies from the process of generating the PE as well as those re-estimated in the target population can be biased due to the method of calculation. The accuracy of GEBV is often estimated as the correlation between the GEBV and a response variable, which can be breeding values, de-regressed proofs, daughter yield deviations, phenotypes or scaled versions of these variables. The co-variances necessary to calculate the correlation are obtained from inner-space vector products of the GEBV vector and the response variable vector [8,10,11]. The expectation of the inner-space vector product of two random vectors with expectation 0 is the trace of their co-variance matrix. Assuming that the co-variance matrix is a matrix times a scalar co-variance, the inner-space vector product will estimate the scalar co-variance correctly only if the average diagonal element of the matrix is 1. If the average is larger than 1, it will inflate the co-variance. Thus, if the covariance matrix between the response variable and the GEBV is the genomic relationship matrix times their covariance, and the average diagonal element of the genomic relationship matrix is larger than 1, the covariance will be biased upwards, and the accuracy will be overestimated. Moreover, genetic trends due to selection may further increase the inner-space vector product due to a mean of the random vectors larger than 0. In addition to the possible bias from transferring GEBV accuracies to the target population and from the method of calculation, the above methodology does not exploit all available phenotypic data when deriving the PE or when estimating the GEBV accuracy. PE using all available data can be derived by a single-step methodology and back-solving the single-step breeding values [27-30]. However, an accuracy in the sense of the proportion of the additive genetic variance explained by GEBV cannot be achieved from such analysis. The REML approach used in this article and by [13-16] overcomes several of the above outlined shortcomings by re-estimating the accuracy in the target population, using as much phenotypic data as available, allowing for sources of bias in parameter estimation due to relationships between individuals, selection and inbreeding, and generating the parameter of paramount interest, the proportion of the genetic variance of the target trait in the target population explained by the genetic covariance between the target trait and the GEBV.

### Across-breed prediction

Across-breed prediction has its theoretical basis in the finding that the LD between SNPs persists over much longer genome distances within breed than across breeds, and in the assumption that trait coding QTL are the same across breeds. Thus, mixing breeds may lead to a sample with most advanced LD decay between QTL and SNPs such that across all breeds in this sample only SNPs in close proximity to the QTL are still in high LD [6,22,31]. Up to this point, this theory is supported by the fact that ALL PE worked best in Angus, followed by ANGUS, BOSTAUR and BRAHMAN PE. However, a consequence of the above logic is that the addition of a breed “N” to a training population of “N-1” breeds must be of decreasing marginal benefit, because the total probability that the LD between QTL and their adjacent SNPs is already exploited increases with every additional individual and/or breed. In practical terms, in a set of “N” breeds, GEBV for breed “N” should be predictable with high accuracy from a training set of “N-1” breeds. Violation of the equal QTL assumption does not invalidate the marginal benefit principle. It also applies when breeds have specific QTL alleles due to mutation or due to an ancestral population with more than two QTL alleles, as long as the LD phase between SNPs and negative/positive QTL alleles is the same for all breeds in the training population. Results in this paper, as well as those published from other across-breed prediction trials [8,14,32], show that all breeds in the target/validation population must be part of the training population to obtain sufficient GEBV accuracies. In the framework of the above theory and its marginal benefit consequence, the conclusion would be that the Limousin and Angus breeds are genetically more different than are the Angus and Brahman breeds, with this difference including different trait coding QTL, inversion of LD phases, fixed SNPs, and SNPs in linkage equilibrium with trait coding QTL. Since such a conclusion contradicts the phylogeny of cattle breeds, the empirical result that across-breed PE yield accurate GEBV only if all targeted breeds are in the training population does not fit into the above genetic theory.

To date, the fact that across-breed prediction works only if all targeted breeds are in the training population may result from partial or total collinearity between very distant SNP genotypes. Collinearity between SNP genotypes can be a result of a physical proximity between two SNPs in terms of base pairs, and can persist over many generations after being induced by an ancient sampling event (e.g. the breed formation). However, collinearity may be also observed between distant SNP genotypes which can be induced by a recent sampling event, for instance sampling a number of individuals for genotyping of which SNP haplotypes do not reflect the genetic diversity in the original population, which is facilitated by the number of SNPs usually exceeding the number of genotyped animals. Such “genotype sampling collinearity” between SNP genotypes in close proximity to a QTL and SNP genotypes very distant from this location will result in LD between QTL and these very distant SNPs. When estimating SNP effects, both types of SNPs will then compete for the effect of the same QTL. Since this kind of collinearity is likely to change with every single individual or breed added to the training population, the prediction equation will change subsequently. How well prediction equations can be transferred to other populations is a function of this change. The empirical finding that targeted breeds must be members of the training population to successfully apply the prediction equation supports the conclusion that much of the LD in current across-breed data sets is induced by the sampling event which arises when individuals are chosen for genotyping.

## Conclusions

Although accuracies of GEBV are generally low compared to already published accuracies that are estimated within breeds, they are in line with those derived from other across-breed prediction trials. Thus, prediction equations derived by the Beef CRC from a mixed-breed training population can contribute to the implementation of genomic selection in Australian beef cattle breeding. Since across-breed prediction equations performed equally or better than the within-breed prediction equations, and the mixed-breed dataset is likely to grow faster than the pure-breed dataset, we recommend that breeders use prediction equations from the mixed-breed training population. However, breeding organisations should only implement GS on the basis of Beef CRC across-breed equations if their breed was part of the training population.
